# Fluorescence lifetime–based multiplex imaging in living plant cells

**DOI:** 10.1093/plphys/kiag475

**Published:** 2026-07-30

**Authors:** Tsuyoshi Aoyama, Nagisa Sugimoto, Yoshikatsu Sato

**Affiliations:** Institute of Transformative Bio-Molecules (WPI-ITbM), Nagoya University, Furo-cho, Chikusa-ku, Nagoya, Aichi 464-8601, Japan; Institute of Transformative Bio-Molecules (WPI-ITbM), Nagoya University, Furo-cho, Chikusa-ku, Nagoya, Aichi 464-8601, Japan; Institute of Transformative Bio-Molecules (WPI-ITbM), Nagoya University, Furo-cho, Chikusa-ku, Nagoya, Aichi 464-8601, Japan; Graduate School of Science, Nagoya University, Furo-cho, Chikusa-ku, Nagoya, Aichi 464-8601, Japan

Dear Editor,

Fluorescence lifetime imaging microscopy (FLIM), which relies on the intrinsic fluorescent lifetime of fluorophores, has become increasingly used. A key advantage of FLIM is its ability to distinguish between fluorophores with similar wavelengths using single-laser excitation, which cannot be achieved by conventional fluorescence intensity imaging based solely on wavelength (Valeur 2001). Fluorescent proteins (FPs) with various fluorescence lifetimes as well as emission spectra have been developed and applied for a multiplex of fluorescently tagged proteins (Murakoshi et al. 2015; Sarkisyan et al. 2015; Mamontova et al. 2018; Mukherjee et al. 2022). However, little is known about which combinations of FPs can be reliably separated based on fluorescence lifetimes, or how large the lifetime difference must be to distinguish between different FPs. Furthermore, although FLIM is expected to be useful especially in plant cells, which exhibit strong autofluorescence in cell walls and chloroplasts, there are still relatively few examples of its application.

To evaluate whether the signals emitted by FPs with similar emission spectra can be separated by FLIM (TCS SP8 FALCON; Leica), we first selected 4 red FPs, namely mCherry, mRFP, mApple, and tdTomato, that possess similar emission spectra and cannot be separated using conventional fluorescence filters (Fig. S1 and Table S1). These red FPs could not be distinguished by conventional intensity-based imaging within the 570 to 620 nm detection range in vitro (Fig. 1a, Fig. S2a to f). However, their signals were successfully separated using FLIM (Fig. 1b, Fig. S2g to l), specifically by phasor plot analysis (Fig. 1c, Fig. S2m to r). A phasor plot visualizes the distribution of fluorescence lifetimes in each pixel as a 2D representation. This approach enables signals with different fluorescence lifetimes to be distinguished without the need for complex multicomponent fitting (Digman et al. 2008; Malacrida et al. 2021). FLIM distinguished any pairwise combination of the 4 FPs, and even all 4 simultaneously (Fig. 1 and Fig. S2). Notably, mCherry and mRFP were readily distinguishable using the phasor plot despite having nearly identical emission spectra (3 nm difference) and very similar fluorescence lifetimes (∼0.2 ns difference) (Fig. 1a, h, o). These results indicate that even small differences in fluorescence lifetime can be resolved by phasor-based FLIM.

**Figure 1:**
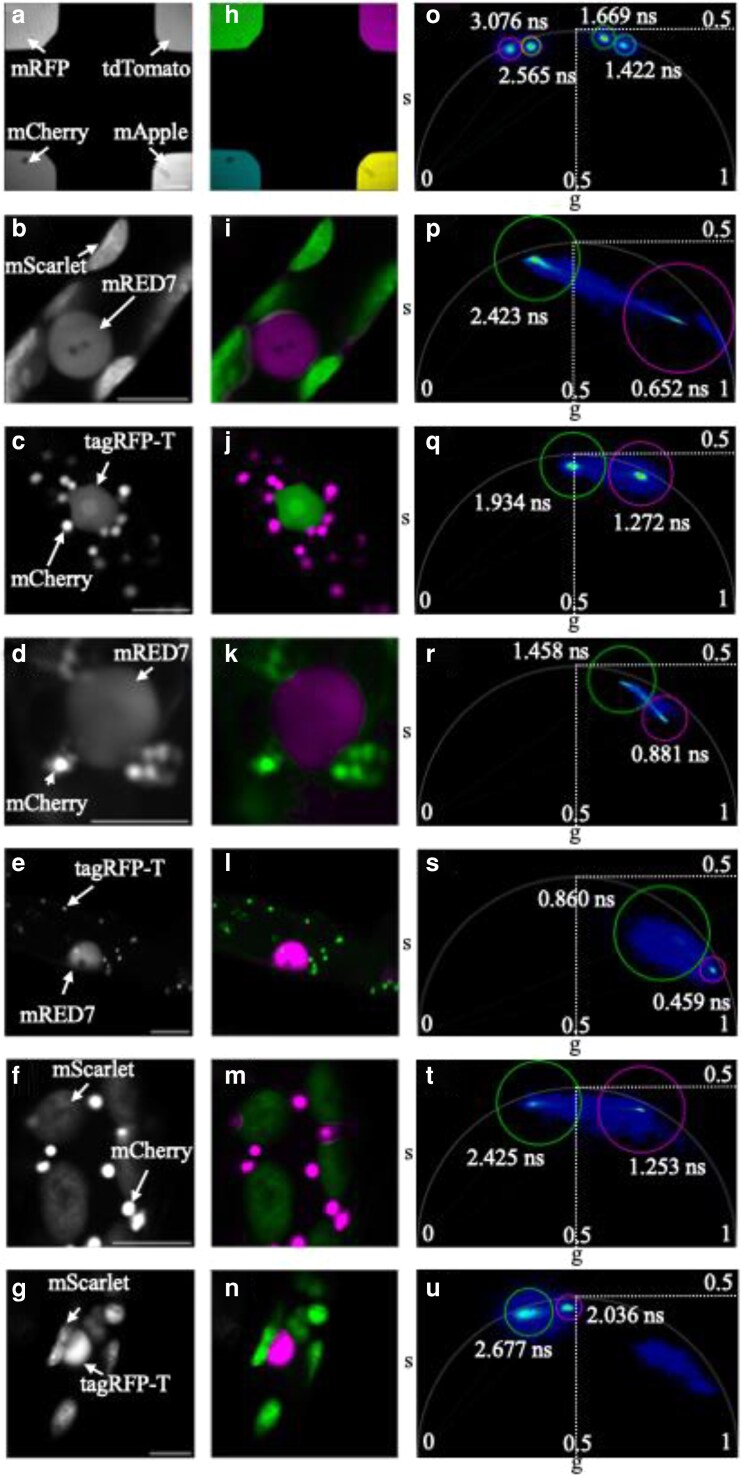
Separation of red FPs based on their fluorescence lifetimes both in vitro and in vivo. a, h, and o) Fluorescence intensity images collected between 570 and 620 nm (a) and pseudo color images (h) based on phasor plot analysis (o) of red FPs (mCherry, mRFP, mApple, and tdTomato). The pseudo colors in (h) correspond to the colors in the phasor plots (o). Each representative image was derived from 3 independent analyses. mCherry, mRFP, mApple, and tdTomato are shown in cyan, green, yellow, and magenta, respectively. Scale bar = 200 μm. b to g, i to n, p to u) Fluorescence intensity images collected between 570 and 620 nm (b to g) and pseudo color images (i to n) based on phasor plot analysis (p to u) of FPs fused with a subcellular localization tag. Tag-fused FPs were transiently expressed in protonemal cells of *P. patens* by particle bombardment. Pseudo colors in (i to n) correspond to the colors in the phasor plots (p to u). b, i, and p) NLS-mRED7 and RBCS-mScarlet are shown in magenta and green, respectively. The representative image set was derived from 9 independent analyses. c, j, and q) mCherry-SKL and NLS-tagRFP-T are shown in magenta and green, respectively. The representative image set was derived from 5 independent analyses. d, k, and r) NLS-mRED7 and mCherry-SKL are shown in magenta and green, respectively. The representative image set was derived from 8 independent analyses. e, l, and s) NLS-mRED7 and tagRFP-T-SKL are shown in magenta and green, respectively. The representative image set was derived from 2 independent analyses. f, m, and t) mCherry-SKL and RBCS-mScarlet are shown in magenta and green, respectively. The representative image set was derived from 6 independent analyses. g, n, and u) NLS-tagRFP-T and RBCS-mScarlet are shown in magenta and green, respectively. The representative image set was derived from 5 independent analyses. Scale bar = 10 μm.

Next, to investigate whether FPs could be simultaneously distinguished in vivo using FLIM, we transiently expressed these proteins in protonemal cells of *Physcomitrium patens* by particle bombardment, fusing each FP with a different subcellular localization tag (Fig. S3, Table S2; Bindels et al., 2017). We analyzed 2 combinations of FPs with different fluorescence lifetime differences: NLS-mRED7 and RBCS-mScarlet (approx. 3.2 ns difference in lifetime), mCherry-SKL and NLS-tagRFP-T (∼0.8 ns difference), respectively. Although intensity-based imaging could not be distinguished from each other (Fig. 1b, c), we observed 2 different populations of signals in the phasor plot (Fig. 1p, q), which corresponded to the predicted localization of each tagged fusion protein (Fig. 1i, j). For other combinations of red FPs, signals with different fluorescence lifetimes were clearly separated using FLIM (Fig. 1k to n, r to u). We also observed 2 combinations of green FPs (GFP, NowGFP and BrUSLEE) in vivo using FLIM. Similar to red FPs, tag-fused green FPs with similar fluorescent wavelengths were also clearly distinguished (Fig. S4). These results indicate that fluorescence lifetime–based multiplex imaging is applicable independent of emission wavelength of the FP.

We next evaluated whether 3 FPs can be separated in vivo using FLIM. When we transiently expressed mCherry-SKL, NLS-tagRFP-T, and RBCS-mScarlet in protonemal cells, fluorescent signals in the peroxisome, nucleus, and chloroplast could not be distinguished by fluorescence intensity imaging, whereas 3 distinct populations with different fluorescence lifetimes were observed in the phasor plot (Fig. 2a, c, f). However, we also observed signals with intermediate fluorescence lifetimes at the boundary regions between peroxisomes and chloroplasts, labeled with mCherry-SKL and RBCS-mScarlet, respectively (Fig. 2c). Similar results were obtained using BrUSLEE-SKL, NLS-GFP, and NowGFP-LTI6b (Fig. 2b, d, g), where not only distinct 3 populations with different fluorescent lifetimes were observed but also signals with intermediate fluorescence lifetimes at the regions between peroxisomes and the plasma membrane, labeled with BrUSLEE-SKL and NowGFP-LTI6b. Mapping a calibration bar onto the phasor plot showed that these signals correspond to fluorescence lifetimes intermediate between those of BrUSLEE and NowGFP (Fig. 2e, h). Similar intermediate lifetimes have been observed for dual-label probes such as FUCCI-red (Shirmanova et al. 2021), where such signals are used for cell cycle analysis. These observations suggest that intermediate lifetime signals can also reflect the relative contributions of different fluorescent species, including in regions of protein co-localization.

**Figure 2:**
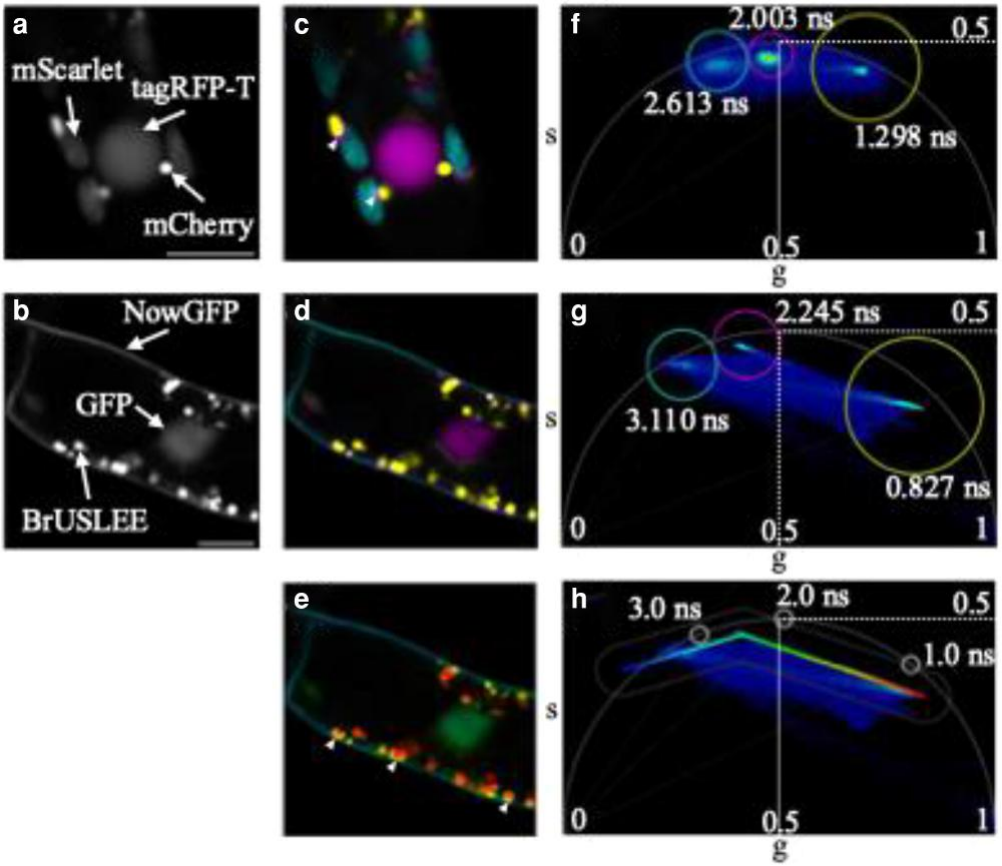
Separation of 3 FPs with similar emission spectra based on their fluorescence lifetimes in vivo. Fluorescence intensity images collected between a) 570 and 620 nm or b) 490 and 540 nm. Pseudo color images (c to e) based on phasor plot analysis (f to h) of FPs fused with a subcellular localization tag. Tag-fused FPs were transiently expressed in protonemal cells of *P. patens* by particle bombardment. Pseudo colors in (c to e) correspond to the colors in the phasor plots (f to h). a, c, and f) mCherry-SKL, NLS-tagRFP-T, and RBCS-mScarlet are shown in yellow, magenta, and cyan, respectively. The arrowheads in (c) indicate intermediate lifetime signals (magenta) detected at the boundary between mCherry-SKL (yellow) and RBCS-mScarlet (cyan) signals. The representative image set was derived from 5 independent analyses. b, d, e, g, and h) BrUSLEE-SKL, NLS-GFP, and NowGFP-LTI6b are shown in yellow, magenta, and cyan, respectively (d and g). BrUSLEE-SKL, NLS-GFP, and NowGFP-LTI6b are shown in red, blue-green, and cyan, respectively (e and h). The arrowheads in (e) indicate intermediate-lifetime signals (yellow) detected at the boundary between BrUSLEE-SKL (red) and NowGFP-LTI6b (cyan) signals. The representative image set was derived from 6 independent analyses. Scale bar, 10 μm.

Although FLIM has traditionally been considered time-consuming, recent advances in TCSPC-based instrumentation have substantially improved acquisition speed. The imaging speed of the current FLIM is now comparable to that of intensity imaging. In this study, we present snapshot images, the fluorescence intensity and FLIM images were acquired simultaneously, and the acquisition time for each image is sufficiently short (eg, pixel dwell time in Fig. 1b, i, p was 3.16 μs and frame rate was 0.193/s for a 512 × 512 pixel image), indicating that multiplex imaging with FLIM should be feasible for time-lapse observations in plant cells. However, careful consideration is required when selecting FPs and determining which tags or proteins they are fused to. While all combinations in this study allowed clear discrimination of the tag-fused FPs, phasor plot analysis showed that tagRFP-T had a shorter fluorescence lifetime in peroxisomes (∼0.9 ns) than in the nucleus (∼2.0 ns) (Fig. 1q, s, u). This difference may reflect local environmental effects, such as variations in pH, viscosity, or ionic composition that influence the photophysical properties of the fluorophore, or alternatively concentration-dependent quenching (homo-FRET). TagRFP-T has been reported to exhibit a tendency to dimerize in vivo (Costantini et al. 2012; Siegel et al. 2013), and increased local concentration within peroxisomes may therefore promote concentration-dependent quenching and reduce fluorescence lifetime. These observations suggest that fluorescence lifetimes can be influenced by the local environment and fluorophore concentration, highlighting the importance of selecting and validating FPs for specific cellular environments in FLIM analyses. Taken together, fluorescence lifetime imaging and conventional wavelength-based fluorescence intensity imaging are orthogonal to each other, allowing them to be combined across different channels. The orthogonality between spectral and fluorescence lifetime information enables multiplex imaging beyond the limits of conventional wavelength-based detection. Combined with ongoing advances in FP engineering, this approach is expected to further expand the flexibility of simultaneous visualization of multiple proteins and cellular processes in living plant cells.

## Materials and methods

### Plant materials and culture conditions

The Cove-NIBB strain of *P. patens* (formerly *Physcomitrella patens*) was used as the wild-type line (Ashton and Cove 1977). Plants were cultured on BCDAT medium (1 mM MgSO_4_, 1.84 mM KH_2_PO_4_, 10 mM KNO_3_, 45 μM FeSO_4_, 5 mM ammonium tartrate, 1 mM CaCl_2_, 0.22 μM CuSO_4_, 10 μM H_3_BO_3_, 0.23 μM CoCl_2_, 0.1 μM Na_2_MoO_4_, 0.19 μM ZnSO_4_, 2 μM MnCl_2_, 0.17 μM KI) solidified with 0.8% agar at 25 ℃ under continuous white light conditions (67.8 μmol m^−2^ s^−1^). For particle bombardment, protonemal tissue was homogenized and cultured on BCDAT agar medium overlaid with cellophane disks for 6 to 7 d.

### Construction of plasmids

Primers used in this study were listed in Table S3. To amplify FP sequence for construction, PIG1b-NLS-GFP-GUS (Ishikawa et al. 2011), His-mCherry pCold, 35S-Lifeact-TagRFP-T pPZP211, and 35S-Lifeact-mScarlet pPZP211 plasmids were used as templates. In addition, mRED7, BrUSLEE-SKL, and NowGFP sequences were synthesized by Integrated DNA Technologies to use as PCR templates. Overexpression vector pT1O, which was modified from the pT1OG (LC126301) by removing gateway cassette rfcA, was used for subcloning. For construction of pT1O-BrUSLEE-SKL, synthesized double-stranded DNA of BrUSLEE-SKL was cloned to pT1O using In-Fusion HD Cloning Kit (Takara). DNA fragments of mCherry-SKL, tagRFP-T-SKL, and NowGFP-SKL were amplified using the following primers: pT1O_mCherry_inf_F, pT1O_mCherry_SKL_inf_R, pT1O_tagRFP-T_inf_F, pT1O_tagRFP-T_SKL_R, pT1O_NowGFP_inf_F, and pT1O_NowGFP_SKL_R. The amplified fragments were cloned to pT1O vector using In-Fusion for construction of pT1O-mCherry-SKL, pT1O-tagRFP-T-SKL, and pT1O-NowGFP-SKL. To generate pT1O-NLS-GFP-mRED7 and pT1O-NLS-GFP-tagRFP-T, DNA fragments of NLS-GFP, mRED7, and tagRFP-T were amplified using the following primers: pT1O_NG_inf_F, NG_mRED7_inf_R, mRED7_F, pT1O_mRED7_inf_R, NLS_tagRFP_inf_R2, tagRFP-T_F, and pT1O-tagRFP-T_R. The amplified fragments were cloned to pT1O vector. To generate pT1O-NLS-BrUSLEE, DNA fragments of NLS and BrUSLEE were amplified using the following primers and cloned to pT1O vector: pT1O_NG_inf_F, NLS_BruSLEE_R, BruSLEE_F, and pT1O_BrUSLEE_R. For construction of RBCS-mScarlet, DNA fragments of RBCS and mScarlet were amplified using the following primers and cloned to pT1O vector: pT1O-RBCS_inf_F, RBCS_mScarlet_inf_R, mScarlet_F, and pT1O-mScarlet_inf_R. DNA fragments of NowGFP and LTI6b were amplified using the following primers and cloned to pT1O vector for construction of NowGFP-LTI6b: pT1O_NowGFP_inf_F, NowGFP_LTI6b_R, LTI6b_inf_F, and LTI6b_pT1O_R.

### Particle bombardment

Particle bombardment experiments were performed using a PDS-1000/He system (Bio-Rad). Gold particles (0.6 μm diameter) were washed with 100% ethanol, rinsed twice with sterile water, and resuspended in sterile water to a concentration of 60 mg/mL. A 10 μL aliquot of the gold particle suspension was mixed with plasmid DNA, followed by the addition of 4 μL of 0.1 M spermidine and 10 μL of 2.5 M CaCl_2_ to precipitate the DNA onto the particles. The amount of plasmid used was adjusted within a range of 200 to 1500 ng, depending on the construct to maintain comparable expression levels. The DNA-coated gold particles were washed with 70% and 100% ethanol and resuspended in 100% ethanol. The suspension was applied to a macrocarrier and allowed to dry. For bombardment, a helium gas pressure of 1,100 psi and a vacuum of 25 in Hg were used. The target shelf was placed at Level 2 (6 cm below the stopping screen). After bombardment, moss tissues were cultured for 1 to 2 d at 25 ℃ under continuous white light conditions (63.5 μmol m^−2^ s^−1^) to allow expression of the transgenes prior to FLIM analysis.

### FLIM analysis

Both intensity and fluorescence lifetime images were acquired using confocal laser scanning microscopy (TCS SP8 FALCON, Leica), an advanced time-correlated single photon counting TCSPC-based system that enables high-speed FLIM, equipped with a pulsed white light laser (80 MHz) and HyD detectors. Using the SP8 FALCON system, fluorescence lifetime and spectral data were collected simultaneously under acquisition settings comparable to those used for conventional confocal imaging. HC PL APO CS2 10×/0.40 and HC PL APO CS2 20×/0.75 objective lenses were used for in vitro and in vivo analysis, respectively. Images were acquired with a pixel dwell time of 3.16 μs and 512 × 512 pixel images. Frame rates were 0.064 to 0.193 s^−1^ depending on frame repetitions (4 to 12). For in vitro analysis, 4 red FPs (mCherry, mRFP, mApple, and tdTomato) used in Kurihara et al. (2021) were diluted in phosphate-buffered saline buffer and loaded into a ViewPlate-1536 (PerkinElmer). Red FPs were excited at 561 nm (2% to 10% of laser power) and detected at 570 to 620 nm. Green FPs were excited at 488 nm (5% to 10% of laser power) and detected at 490 to 540 nm. FLIM data were analyzed using the LAS X (Leica) software suite by either phasor plot analysis or exponential-fitting. For phasor analysis, lifetime values were calculated automatically by LAS X with the harmonic parameter set to 1. For fitting analysis, the n-Exponential Tail Fit model was used with a single-exponential component. Calculated lifetimes for each experiment and the results of the χ^2^ test are reported in Table S1.

## Supplementary Material

kiag475_Supplementary_Data

## Data Availability

The data underlying this article are available in the article and its online supplementary material. Additional data, including plasmid sequences, are available from the corresponding author upon reasonable request.
