# Supplementary material for: Fluorescence lifetime–based multiplex imaging in living plant cells
Source: Plant Physiol. 2026 Jul 30;201(3):kiag475. doi: 10.1093/plphys/kiag475 (PMC13421888; doi:10.1093/plphys/kiag475)
Supplement: kiag475_Supplementary_Data [file kiag475_supplementary_data.zip › Supplemental Figures.pdf]

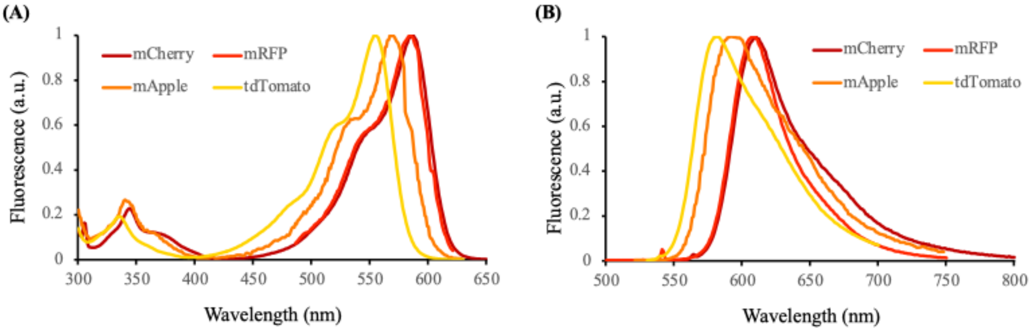

Supplemental Figure S1. Excitation and emission spectra of red fluorescent proteins

(A) Excitation spectra and (B) emission spectra of mCherry, mRFP, mApple, and tdTomato. Spectra are normalized to the maximum intensity. Spectral data for each fluorescent protein were obtained from FPbase (https://www.fpbase.org).

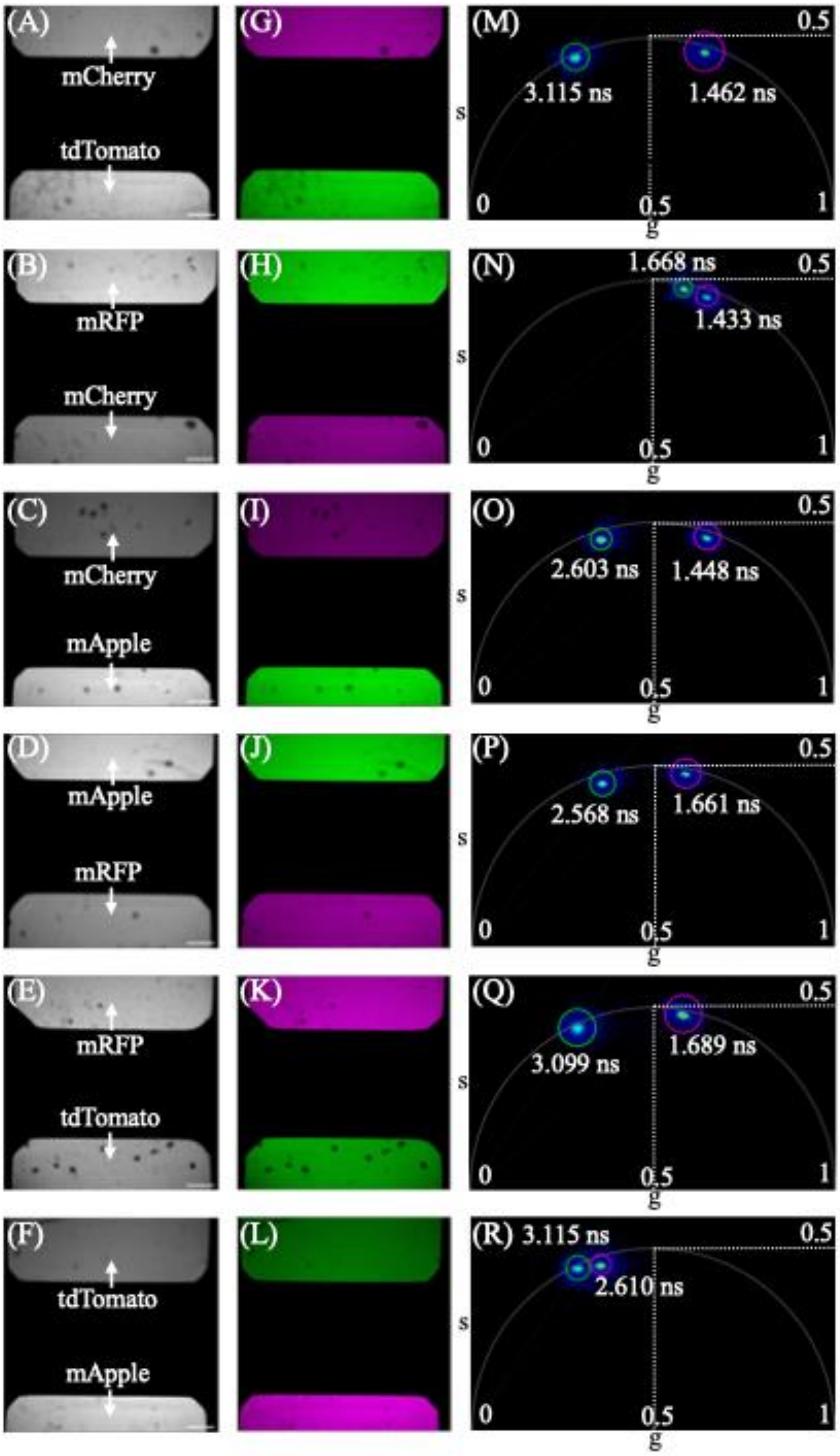

Supplemental Figure S2. Separation of red fluorescent proteins based on their fluorescence lifetimes *in vitro*.

Fluorescence intensity images collected between 570-620 nm (A-F) and pseudo color images (G-L) based on phasor plot analysis (M-R) of red fluorescent proteins (mCherry, mRFP, mApple, and

tdTomato). The pseudo colors in (G-L) correspond to the colors in the phasor plots (M-R). Each representative image was derived from three independent analyses. **A, G, M** mCherry and tdTomato are shown in magenta and green, respectively. **B, H, N** mCherry and mRFP are shown in magenta and green, respectively. **C, I, O** mCherry and mApple are shown in magenta and green, respectively. **D, J, P** mRFP and mApple are shown in magenta and green, respectively. **E, K, Q** mRFP and tdTomato are shown in magenta and green, respectively. **F, L, R** mApple and tdTomato are shown in magenta and green, respectively. Scale bar = 200 μm.

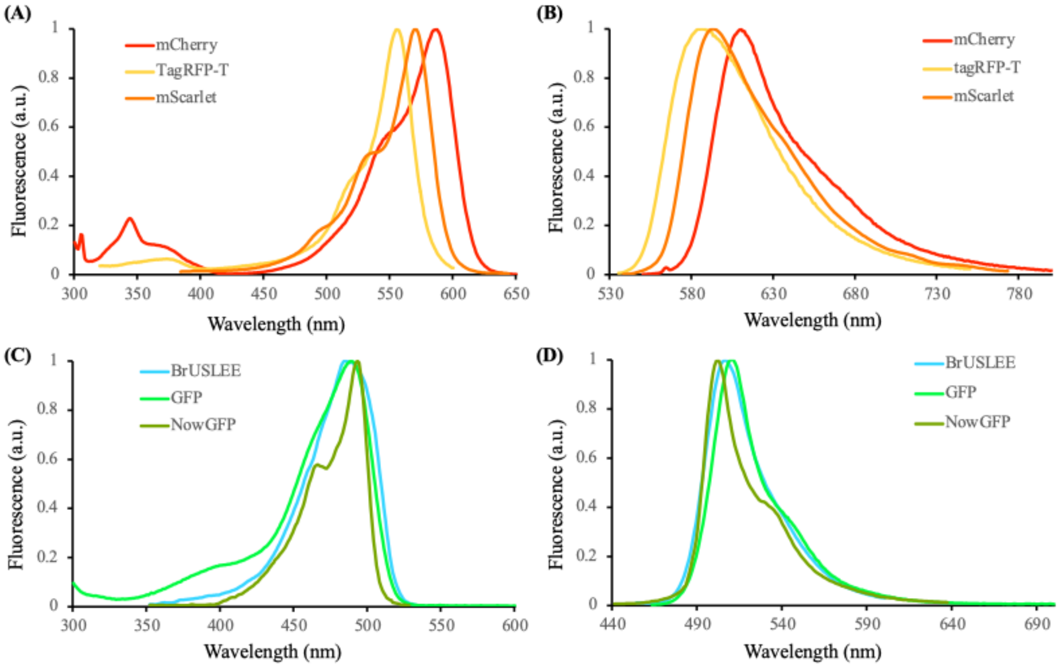

Supplemental Fig. S3

Supplemental Figure S3. Excitation and emission spectra of fluorescent proteins used in *in vivo* analysis

A–B Excitation (A) and emission (B) spectra of mCherry, TagRFP-T, and mScarlet. **C–D** Excitation (C) and emission (D) spectra of BrUSLEE, GFP, and NowGFP. Spectra are normalized to the maximum intensity. Spectral data were obtained from FPbase (https://www.fpbase.org).

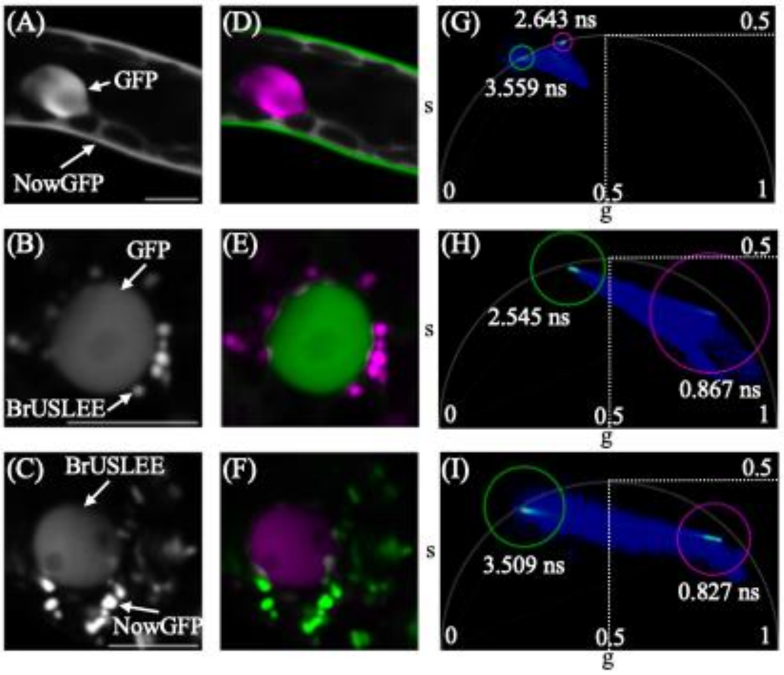

Supplemental Figure S4. Separation of green fluorescent proteins based on their fluorescence lifetimes *in vivo*.

Fluorescence intensity images collected between 490-540 nm (A-C) and pseudo color images (D-F) based on phasor plot analysis (G-I) of fluorescent proteins fused with a subcellular localization tag. Tag-fused fluorescent proteins were transiently expressed in protonemal cells of *P. patens* by particle bombardment. Pseudo colors in (D-F) correspond to the colors in the phasor plots (G-I). **A, D, G** NLS-GFP and NowGFP-LTI6b are shown in magenta and green, respectively. The representative image set was derived from 6 independent analyses. **B, E, H** BrUSLEE-SKL and NLS-GFP are shown in magenta and green, respectively. The representative image set was derived from 7 independent analyses. **C, F, I** NLS-BrUSLEE and NowGFP-SKL are shown in magenta and green, respectively. The representative image set was derived from 5 independent analyses. Scale bar: 10 μm
