# Supplementary material for: Fluorescence lifetime–based multiplex imaging in living plant cells
Source: Plant Physiol. 2026 Jul 30;201(3):kiag475. doi: 10.1093/plphys/kiag475 (PMC13421888; doi:10.1093/plphys/kiag475)
Supplement: kiag475_Supplementary_Data [file kiag475_supplementary_data.zip › Supplemental_Tables.docx]

Supplemental Table1. Red fluorescent proteins used for the *in vitro* experiments

| Name | Exλ  (nm) | Emλ  (nm) | Lifetime*  (ns) | Lifetime in this study**  (ns) | χ²** |
| --- | --- | --- | --- | --- | --- |
| mCherry | 587 | 610 | 1.5 | 1.509 | 1.062 |
|  |  |  |  | 1.512 | 0.952 |
|  |  |  |  | 1.542 | 1.291 |
| mRFP | 584 | 607 | n.a. | 1.725 | 0.957 |
|  |  |  |  | 1.724 | 0.846 |
|  |  |  |  | 1.751 | 0.748 |
| mApple | 568 | 592 | 2.9 | 2.773 | 0.890 |
|  |  |  |  | 2.780 | 0.951 |
|  |  |  |  | 2.768 | 0.815 |
| tdTomato | 554 | 581 | 3.4 | 3.282 | 0.927 |
|  |  |  |  | 3.254 | 0.866 |
|  |  |  |  | 3.216 | 0.712 |

* These fluorescent lifetime values were taken from Bindels DS et al. 2017.

** Purified proteins dissolved in PBS buffer were used. Fluorescence lifetimes and χ² values were calculated by fitting the data using the n-exponential tail-fit model implemented in SP8 FALCON. Experiments were performed in triplicate.

Supplemental Table2. Information of fluorescent proteins used for the *in vivo* experiments

| Name | Exλ (nm) | Emλ (nm) | Lifetime (ns) | Reference for FP |
| --- | --- | --- | --- | --- |
| mRED7 | 589 | 606 | 0.7 | Bindels DS et al. 2017 |
| mCherry | 587 | 610 | 1.5 | Bindels DS et al. 2017 |
| tagRFP-T | 555 | 584 | 2.3 | Bindels DS et al. 2017 |
| mScarlet | 569 | 594 | 3.9 | Bindels DS et al. 2017 |
| BrUSLEE | 487 | 509 | 0.82 | Mamontova et al. 2018 |
| GFP | 488 | 507 | 2.8 | Mamontova et al. 2018 |
| NowGFP | 494 | 502 | 5.1 | Sarkisyan et al. 2015 |

Supplemental Table3. Primers used in this study

| Primer name | Sequence (5’ to 3’) |
| --- | --- |
| pT1O_mCherry_inf_F | AGGTAATCGAAGGTTATGGTGAGCAAGGGCGAGGAGGATA |
| pT1O_mCherry_SKL_inf_R | ATCTACAGGCCTGTTTTATAGTTTCGACTTGTACAGCTCGTCCATGCCG |
| pT1O_tagRFP-T_inf_F | AGGTAATCGAAGGTTATGGTATCAAAAGGGGAGGAATTAATTAAA |
| pT1O_tagRFP-T_SKL_R | ATCTACAGGCCTGTTTTATAGTTTCGAATTGAGCTTATGTCCTAACTTA |
| pT1O_NowGFP_inf_F | AGGTAATCGAAGGTTATGGTCTCAAAGGGGGAGAAGCTTTTTACA |
| pT1O_NowGFP_SKL_R | ATCTACAGGCCTGTTTTATAGTTTCGACTTGTACAGTTCATCGGCTCCC |
| pT1O_NG_inf_F | AGGTAATCGAAGGTTATGGCTGATCCAAAAAAGAAGAGAA |
| NG_mRED7_inf_R | ACCTTTACTTACCATAAGGGACTGACCACCCGCTGCTCCT |
| mRED7_F | ATGGTAAGTAAAGGTGAGGAAGTCA |
| pT1O_mRED7_inf_R | ATCTACAGGCCTGTTTCACTTGTATAACTCGTCCATCCCC |
| NLS_tagRFP_inf_R2 | CCCTTTTGATACCATAAGGGACTGACCACCCGCTGCTCCTGCTCC |
| tagRFP-T_F | ATGGTATCAAAAGGGGAGGAATTAATTAAA |
| pT1O-tagRFP-T_R | ATCTACAGGCCTGTTTCAATTGAGCTTATGTCCTAACTTACTGGG |
| NLS_BruSLEE_R | TCCTTTAGAGACCATCCCATCTACCTTTCTCTTCTTTTTTGGATC |
| BruSLEE_F | ATGGTCTCTAAAGGAGAGGAGTTATTCACT |
| pT1O_BrUSLEE_R | ATCTACAGGCCTGTTTTATTTGTATAATTCGTCCATCCCCAAGGT |
| pT1O-RBCS_inf_F | AGGTAATCGAAGGTTATGGCTTCCTCTATGTTCTCCTCCA |
| RBCS_mScarlet_inf_R | TCCCTTTGATACCATAGCTTCGGTGAAGCTTGGGGGCTTG |
| mScarlet_F | ATGGTATCAAAGGGAGAGGCCGTGA |
| pT1O-mScarlet_inf_R | ATCTACAGGCCTGTTTCACTTATACAACTCATCCATACCCCCT |
| NowGFP_LTI6b_R | TCCACCACCTCCAGACTTGTACAGTTCATCGGCTCCCAACGGGAT |
| LTI6b_inf_F | TCTGGAGGTGGTGGATCTGGAGGTGGTGGAGCCATGGGTA |
| LTI6b_pT1O_R | ATCTACAGGCCTGTTTCACTTGGTGATGATATAAAGAGCG |
